# Development and evaluation of the first high-throughput SNP array for common carp (*Cyprinus carpio*)

**DOI:** 10.1186/1471-2164-15-307

**Published:** 2014-04-24

**Authors:** Jian Xu, Zixia Zhao, Xiaofeng Zhang, Xianhu Zheng, Jiongtang Li, Yanliang Jiang, Youyi Kuang, Yan Zhang, Jianxin Feng, Chuangju Li, Juhua Yu, Qiang Li, Yuanyuan Zhu, Yuanyuan Liu, Peng Xu, Xiaowen Sun

**Affiliations:** 1 Centre for Applied Aquatic Genomics, Chinese Academy of Fishery Sciences, Beijing 100141, China; 2 Heilongjiang Fisheries Research Institute, Chinese Academy of Fishery Sciences, Harbin 150070, China; 3 Henan Academy of Fishery Sciences, Zhengzhou, Henan 450044, China; 4 Yangtze River Fisheries Research Institute, Chinese Academy of Fishery Sciences, Wuhan 430223, China; 5 Freshwater Fisheries Research Center, Chinese Academy of Fishery Sciences, Wuxi 430223, China; 6 Visiting Professor Department of Zoology, College of Science, King Saud University, P. O. Box 24555, Riyadh 11451, Saudi Arabia

**Keywords:** SNP array, Affymetrix, Re-sequencing, Linkage disequilibrium, Identity by state, *Cyprinus carpio*, Common carp, Cyprinidae

## Abstract

**Background:**

A large number of single nucleotide polymorphisms (SNPs) have been identified in common carp (*Cyprinus carpio*) but, as yet, no high-throughput genotyping platform is available for this species. *C. carpio* is an important aquaculture species that accounts for nearly 14% of freshwater aquaculture production worldwide. We have developed an array for *C. carpio* with 250,000 SNPs and evaluated its performance using samples from various strains of *C. carpio*.

**Results:**

The SNPs used on the array were selected from two resources: the transcribed sequences from RNA-seq data of four strains of *C. carpio*, and the genome re-sequencing data of five strains of *C. carpio*. The 250,000 SNPs on the resulting array are distributed evenly across the reference *C.carpio* genome with an average spacing of 6.6 kb. To evaluate the SNP array, 1,072 *C. carpio* samples were collected and tested. Of the 250,000 SNPs on the array, 185,150 (74.06%) were found to be polymorphic sites. Genotyping accuracy was checked using genotyping data from a group of full-siblings and their parents, and over 99.8% of the qualified SNPs were found to be reliable. Analysis of the linkage disequilibrium on all samples and on three domestic *C.carpio* strains revealed that the latter had the longer haplotype blocks. We also evaluated our SNP array on 80 samples from eight species related to *C. carpio*, with from 53,526 to 71,984 polymorphic SNPs. An identity by state analysis divided all the samples into three clusters; most of the *C. carpio* strains formed the largest cluster.

**Conclusions:**

The Carp SNP array described here is the first high-throughput genotyping platform for *C. carpio*. Our evaluation of this array indicates that it will be valuable for farmed carp and for genetic and population biology studies in *C. carpio* and related species.

## Background

Common carp (*Cyprinus carpio*) is naturally distributed across Europe and Asia. It was domesticated about 2,000 years ago, and is cultured in over 100 countries worldwide with over 3 million metric tons of global annual production [1,2]. As a result of selection and breeding efforts over the past centuries, many domesticated strains have been established with distinct economic traits or phenotypes adapted to local environments and to meet consumer demands. China is the largest *C. carpio* producer, and there are abundant domesticated strains and populations in China, including Sonpu mirror carp, Hebao red carp, Xingguo red carp, Yellow River carp, and Oujiang color carp, as well as many hybrid strains, all of which are the basis and genetic resources for selective breeding using modern genetic tools.

Because of the economic importance of *C. carpio* for the global aquaculture industry, as well as its importance as a model species for ecology, physiology, and evolutionary studies, over the past decade, researchers have developed a variety of genetic and genomics tool and resources. A large number of genetic markers have been developed, including microsatellites [3,4], and single nucleotide polymorphisms (SNPs) [5,6]. A number of genetic linkage maps have been constructed based on these markers [7-10]. The markers have also been used to identify quantitative trait loci (QTLs) associated with economically important traits including growth rate, body shape, and meat quality [4,11,12]. A large set of expressed sequence tags (ESTs) have been generated using traditional cloning and Sanger sequencing methods, or next-generation transcriptome sequencing, and a cDNA microarray has been designed and constructed [13-17]. A bacterial artificial chromosome (BAC) library has been built [18], a BAC-based physical map has been constructed, and a large set of BAC-end sequences (BES) have been generated [19,20]. The complete mitochondrial genomes of several strains and populations have been sequenced [21-23]. Whole genome exome data were generated for a comparative study with the *Danio rerio* genome [24] and, recently, the *C. carpio* genome consortium has completely sequenced and assembled a draft genome sequence of *C. carpio*[25].

A major gap in the *C. carpio* toolkit is the lack of a high-throughput SNP genotyping platform for genetic research. Such a platform is essential for whole genome association studies (GWAS) of important traits, as well as for genome-assisted selection in breeding programs. Genome-scale SNP genotyping is most efficiently performed using SNP arrays or chips. Arrays of this type have been used widely in genetic studies in humans, as well as in important model organisms and agriculture species.

The reductions in the cost of acquiring sequence data using next-generation sequencing technologies has led to the development of genotyping by sequencing (GBS) approaches, which use whole genome sequencing, reduced representative genome sequencing, or target-enriched DNA sequencing data to determine genotypes. The most popular GBS protocol is restriction-site-associated DNA (RAD) tag sequencing in which DNA fragments flanking particular restriction sites are targeted for sequencing, thereby allowing the discovery and genotyping of SNPs at these targeted locations [26]. Although GBS methods have some advantages for genome-wide SNP discovery and genotyping, especially for species for which a reference genome has not been established, they also have limitations, which include the requirements for complicated DNA library preparation procedures and intensive bioinformatics pipelines. GBS is not suitable for genotyping the very large numbers of individuals or SNP loci that are used commonly in GWAS and genomic selection. In addition, GBS genotyping results are not shared easily among different research groups because the same SNP loci are not assayed in all individuals.

Therefore high-density SNP genotyping arrays remain the tools of choice for high-resolution genetics analysis. Many SNP arrays or chips have been developed for either Illumina or Affymetrix platforms, including the human 500 K array, the Genome-Wide Human SNP Array 5.0 and 6.0, the porcine 60 K SNP array [27], the bovine 50KSNP array [28], the chicken 60 K [29] and 600 K SNP arrays [30], the canine 22 k SNP array [31], and the equine 50 K SNP array [32]. These arrays have been used widely for research on selective sweeps, phylogeny, population structure, copy number variations, GWAS, and other aspects [32-36], boosting genome and genetic studies as well as breeding programs of these species.

Although the importance of high-density SNP genotyping arrays has been recognized widely, as yet there are only a few such SNP genotyping arrays for aquaculture species. After the submission of this manuscript, an Affymetrix Axiom® myDesign Custom Array containing 132,033 Atlantic salmon SNPs was developed [37]. Meanwhile, an Affymetrix Axiom Array containing 204,437 putative catfish SNPs was also developed [38]. Although a large research community is working on *C. carpio* and other closely related Cyprinid species, and genotyping is performed intensively for diverse purposes, no SNP genotyping array is available for *C. carpio*.

Here, we report the design and validation of the first high-density *C. carpio* SNP array, the Carp SNP array, based on the Affymetrix Axiom platform. The Carp SNP array was validated with 1,072 samples from various *C. carpio* populations and strains. To assess its potential use in closely related Cyprinids, we also validated the array in 80 individuals from eight related species. A pilot study was conducted to demonstrate the accuracy and efficiency of the genome-scale genotyping and linkage disequilibrium (LD) decay was analyzed in all samples and in several domesticated strains. Identity by state (IBS) clustering of all samples was conducted, which demonstrated the reliability of the Carp SNP array.

## Results and discussion

**Figure 1:**
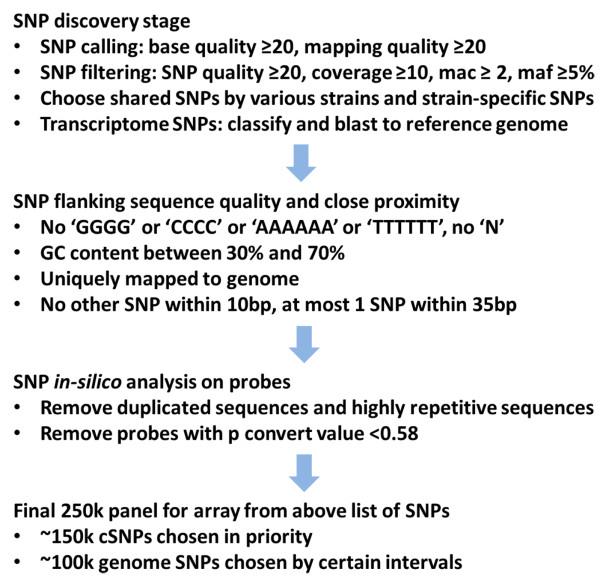
Pipeline of carp SNP array development.

The pipeline and design parameters described below are summarized in Figure 1.

### Sequencing and alignment of sequence reads

In previous studies, over 700,000 SNPs have been identified in transcript sequences and classified [5]. All these SNPs were mapped to the reference genome and assigned to genomic positions. However, because these SNPs are from transcribed sequences, their numbers are limited and represent only the SNPs in coding sequences. To improve on this situation, we selected 18 representative carps for genome re-sequencing, including seven accessions of two wild populations from the Yellow and Heilongjiang rivers, and 11 accessions of three domesticated strains (Songpu, Oujiang color, and Hebao). Re-sequencing of these 18 accessions generated a total of 2,281 million paired-end reads that were 101 bp long (228.1 Gb). All raw sequencing data have been deposited in the NCBI Sequence Read Archive [SRA: SRP026407]. The short reads were mapped to the reference genome, with an average sequencing depth of six genome equivalent per animal. The mapping coverage rate was an average of 87.6% (Table 1).

**Table 1:** Genome re-sequencing data

**Accession**	**Raw bases (G)**	**Mapped bases (G)**	**Mapping rate (%)**	**Coverage rate (%)**	**Depth**
Songpu carp 1	11.71	9.90	84.54	89.32	5.84
Songpu carp 2	12.39	10.40	83.93	89.09	6.14
Songpu carp 3	12.66	10.64	84.10	89.35	6.28
Songpu carp 4	11.45	9.62	84.02	88.58	5.68
Yellow River carp 1	10.40	8.30	79.87	86.62	4.90
Yellow River carp 2	10.43	8.33	79.84	87.04	4.91
Yellow River carp 3	11.92	9.08	76.16	87.19	5.36
Yellow River carp 4	14.01	11.11	79.31	89.18	6.56
Heilongjiang River carp 1	13.40	9.21	68.75	86.73	5.44
Heilongjiang River carp 2	16.13	12.25	75.93	89.72	7.23
Heilongjiang River carp 3	15.22	9.46	62.20	87.49	5.58
Hebao carp 1	9.86	7.92	80.32	85.60	4.67
Hebao carp 2	13.63	9.53	69.90	85.63	5.62
Hebao carp 3	13.07	10.36	79.23	88.28	6.11
Hebao carp 4	12.54	10.21	81.44	88.11	6.03
Oujiang color carp 1	11.98	9.65	80.52	87.00	5.69
Oujiang color carp 2	11.39	8.23	72.27	85.65	4.86
Oujiang color carp 3	10.66	8.26	77.48	85.63	4.87

### SNP identification

SNP identification was performed separately within each strain. The criteria used for calling SNPs were as following: (1) mapping quality score ≥ 20; (2) relevant base quality score ≥ 20; (3) SNP quality score ≥ 20 and SNP position must be covered by at least 10 reads; and (4) minor allele count (MAC) ≥ 2 and minor allele frequency (MAF) ≥ 5%. A total of 8,058,251 SNPs were identified in Songpu carp, 11,412,638 SNPs in Yellow River carp, 8,688,799 SNPs in Heilongjiang River carp, 7,123,672 SNPs in Oujiang color carp, and 9,955,915 SNPs in Hebao carp (Table 2). Overall, a total of 24,272,905 non-redundant SNPs were identified, of which 802,209 were shared by all strains, and 13,811,200 were strain-specific. Together with the SNPs identified previously in the transcript sequences, we had a pool of 15,366,108 SNPs from which to select SNPs for the carp array. An abundant source of candidate SNPs is essential for designing SNP arrays, especially for large genomes like the *C. carpio* genome. When the dog SNP array was developed, more than 2.5 million potential SNPs were identified, with one SNP per 0.9 kb between breeds and one SNP per 1.5 kb within breeds. In other studies, 2.8 million SNPs were detected in chicken [9], and 1.1 million SNPs were discovered in horse [36]. Thus, based on these previous studies, it is evident that we had gathered a sufficient number of candidate SNPs to develop a *C. carpio* SNP array.

**Table 2:** SNP identification from genome re-sequencing

**Strain**	**No. SNPs**	**No. strain-specific**	**No. shared**
Songpu mirror carp	8,058,251	2,434,141	
Yellow River carp	11,412,638	3,674,888	
Heilongjiang River carp	8,688,799	2,337,049	802,209
Oujiang color carp	7,123,672	2,209,060	
Hebao carp	9,955,915	3,156,062	
Non-redundant	24,272,905		

### SNP reduction based on flanking sequence quality and close proximity

For quality control, 71-bp fragments spanning each SNP were extracted, including 35-bp upstream and 35-bp downstream of the SNP base. SNPs with flanking sequences that containing over four consecutive ‘G’ or ‘C’ or over six consecutive ‘A’ or ‘T’, and those containing ‘N’ were removed, resulting in 13,431,573 SNPs. Next, GC content was calculated and SNPs with flanking sequences with GC content below 30% or above 70% were removed. The flanking sequences of the remaining 11,307,040 SNPs were mapped to the reference genome, and the 8,450,637 SNPs that mapped uniquely were kept for further selection. SNPs located very close to each other are less likely to be assayed successfully during genotyping because of interference from neighboring variants. Clustering of SNPs can be a result of the mis-alignment of reads because of the presence of the indels (insertions or deletions) at the beginning or end of reads [39]. Based on advice from Affymetrix scientists, we removed SNPs that were within 10 bp of each other or there were more than two variants within 35 bp. After these steps, 3,719,260 SNPs remained in the final pool for selection. Priority was given to SNPs in coding sequences, and then the genome re-sequencing SNPs were selected on the basis of their quality scores and spacing on the genome. Finally, a total of 378,815 SNPs were submitted for probe design.

### SNP reduction based on *in-silico* analysis of conversion values

**Figure 2:**
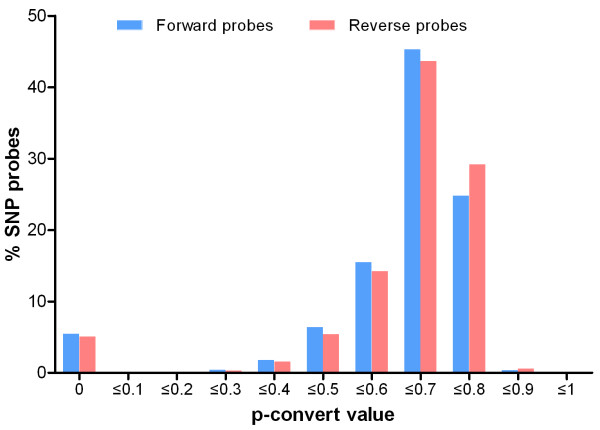
P-convert value for candidate probes.

The 378,815 selected SNPs were submitted to Affymetrix for *in-silico* analysis to predict their reproducibility on the Axiom platform. The p -convert value, which is calculated using a random forest model, is designed to predict the probability that the SNP will convert on the array. The random forest model considers many factors, such as probe sequence, binding energies, unexpected non-specific binding and probability of hybridization to multiple genomic regions [30]. P-convert values were generated for the forward and reverse probes and p-convert values ≥ 0.58 were considered to be qualified. As shown in Figure 2, a high proportion of the 378,815 SNPs (347,712; 91.8%) had a p-convert value ≥ 0.58.

### SNP selection for the final Carp array

**Figure 3:**
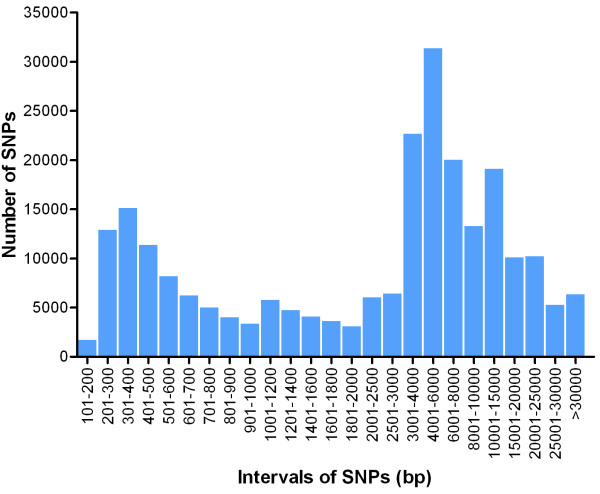
Distribution of intervals of array SNPs.

**Figure 4:**
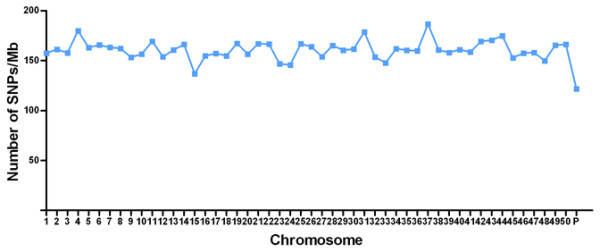
**Densities of SNPs over 50 chromosomes and unassembled scaffolds.** Densities of SNPs were calculated on 50 chromosomes by a unit of 1 million base pair. SNPs on unassembled scaffolds were joined together to form a pseudo “P” chromosome.

In this final step, we selected 250,000 SNPs in the following order: (1) 8,204 non-synonymous SNPs and 5,219 SNPs in UTR regions with each SNP at least 100 bp from any adjacent SNP; (2) 133,603 SNPs in transcribed sequences that were at least 1.8 kb from any adjacent selected SNP; (3) 100,974 SNPs from the genome re-sequencing data that were shared between strains and separated by at least 10 kb from any adjacent selected SNP; and (4) 2,000 strain-specific SNPs that were at least 17 kb from any other SNP on the array (Table 3). As shown in Figure 3, the average interval between the final 250,000 SNPs was 6.6 kb, and the intervals between most SNPs ranged from 3 to 8 kb. When the SNP densities on the assembled *C. carpio* chromosomes were calculated, we found that the SNP densities ranged from 137 sites/Mb to 187 sites/Mb. Scaffolds that have not been assigned to one of the 50 chromosomes were joined to form a pseudo ‘P’ chromosome, which had a SNP density of 122 sites/Mb (Figure 4). Thus, the average number of SNPs per unit physical distance indicates that the SNPs are uniformly distributed across the genome.

**Table 3:** Number of SNPs during SNP array designation

**Category**	**Original**	**Stage 1**					**Stage 2**	
		**Repetitive nucleotides**	**GC content**	**Unique mapping**	**Adjacent SNPs**	**Pre-screen**	**Probe QC**	**Final**
Transcriptome sequencing								147,026
Non-synonymous	47,137	32,489	32,315	25,211	11,813	9,669	8,204	8,204
3′UTR	19,639	13,734	12,758	11,314	5,340	3,819	3,616	3,616
5′UTR	8,145	6,488	6,420	5,042	2,516	1,864	1,603	1,603
Others	670,325	629,039	586,832	475,850	220,137	155,437	140,879	133,603
Genome re-sequencing								102,974
Strain-shared	809,662	745,423	660,045	532,121	213,189	168,216	157,579	100,974
Strain-specific	13,811,200	12,004,400	10,008,670	7,401,099	3,266,265	39,810	35,831	2,000
Total	15,366,108	13,431,573	11,307,040	8,450,637	3,719,260	378,815	347,712	250,000

### Evaluation of the SNP array in *C. carpio* strains

After the Carp array was manufactured, we evaluated the array in both *C. carpio* and related carp species. A total of 1,072 *C. carpio* samples were collected from various strains, including Songpu carp, Hebao carp, Yellow River carp, Oujiang color carp, Xingguo red carp, and Heilongjiang carp. Of the 250,000 candidate SNPs, 223,274 (89.3%) passed the manufacturing quality control and could be genotyped. With a stringent call rate threshold of 95%, there were 185,150 (74.06%) polymorphic sites, 4,202 (1.68%) sites with no minor homology genotype, 180 (0.07%) monomorphic sites, and 33,742 (13.50%) sites below the call rate threshold (Table 4). Although 185,150 (74.06%) polymorphic SNPs had been validated in this study, it does not mean that only 185,150 loci are polymorphic. More SNP loci will be validated when more strains harboring a new genetic background are genotyped using this array. Genotyping accuracies were estimated using samples from families and the results seemed to be satisfactory (data not shown). Of the189,532 SNPs that passed the call rate threshold, 80.0% had a MAF > 0.10 and 63.3% had a MAF > 0.20, indicating that most of the SNPs will be applicable in subsequent research.

**Table 4:** Evaluation of SNP array in all samples

**Category**	***C. carpio***			**Related species of *C. carpio***		
	**Percentage (%)**	**SNP count**	**Probe count**	**Percentage (%)**	**SNP count**	**Probe count**
Poly high resolution	74.06	185,150	220,615	21.65	54,116	60,143
No minor homology	1.68	4,202	7,173	2.70	6,748	8,772
Mono high resolution	0.07	180	315	0.04	88	126
Call rate below threshold	13.50	33,742	58,146	9.59	23,981	32,564
Off Target Variation (OTV)	1.15	3,610	3,610	4.38	10,941	20,424
Other	9.54	23,844	26,734	61.65	154,126	194,564
Total	100.00	250,000	316,593	100	250,000	316,593

### Accuracy of genotyping for the SNP array

High accuracy is a vital parameter for a genotyping platform. In this study, we assessed the genotyping accuracy of our Carp array using data from a family comprising two parents and 80 offspring. PLINK software was applied with the ‘Mendel’ parameter. Any genotypes not concordant between parents and offspring were regarded as genotyping errors. We estimated the accuracy to be 99.6% on average, and after excluding one sample because of multiple inconsistencies with the inheritance pattern expected on the basis of the declared pedigree, the genotyping accuracy increased to 99.8% on average, showing the high genotyping quality of the Carp array. Thus, in subsequent research, this array will be of great importance in trait association analysis, QTL mapping, and marker assisted selection.

### Extensive assessment of the SNP array in Cyprinids

We evaluated the SNP array in 80 samples from the *C. carpio* related species, such as *Carassius carassius, Ctenopharyngodon idella*, *Mylopharyngodon piceus, Hypophthalmichthys molitrix, Hypophthalmichthys nobilis, Megalobrama amblycephala, Danio rerio, Leuciscus waleckii*, and 84,933 (34.0%) SNPs were found to be polymorphic. With a moderate call rate threshold of 80%, there were 54,116 (21.65%) polymorphic sites, 6,748 (2.70%) sites with no minor homology genotype, 88 (0.04%) monomorphic sites, and 23,981 (9.59%) sites below the call rate threshold (Table 4). A detailed analysis of the eight Cypridinae species is shown in Table 5. The number of SNPs that exhibited variations for each species ranged from 53,526 to 71,984, demonstrating that the SNP array is potentially useful for studies of carp-related species. After filtering the SNP call rate, the remaining number of SNPs range from 29,870 to 59,020 among the eight species. The significant difference in the SNP numbers before and after filtering is mainly because of the small sample sizes. From the eight Cypridinae species, we collected 15 samples of *D. rerio*, five samples of *L. waleckii*, and 10 samples for other six species. In future research, as large numbers of samples are collected, more of the SNPs on the array may pass the call-rate threshold. Among these eight species, *D. rerio* is the only species for which a genome assembly has been reported.

**Table 5:** **Evaluation of SNP array in eight *Cyprinus carpio *** **related species**

**Category**	**SNP count**							
	***C. carassius *** **(n = 10)**	***M. piceus *** **(n = 10)**	***C. idella *** **(n = 10)**	***H. molitrix *** **(n = 10)**	***H. nobilis *** **(n = 10)**	***M. amblycephala *** **(n = 10)**	***D. rerio *** **(n = 15)**	***L. waleckii *** **(n = 5)**
Poly high resolution	17,447	30,872	8,162	9,153	18,629	18,816	2,911	1,556
No minor homology	30,581	28,148	27,880	27,514	28,623	31,707	37,499	28,314
Mono high resolution	0	0	0	0	0	0	0	0
Call rate below threshold	22,240	12,964	19,192	19,230	18,532	16,751	14,533	23,656
Off Target Variation (OTV)	10,941	10,941	10,941	10,941	10,941	10,941	10,941	10,941
Other	168,791	167,075	183,825	183,162	173,275	171,785	184,116	185,533
Total	250,000							

### Linkage disequilibrium (LD) analysis

**Figure 5:**
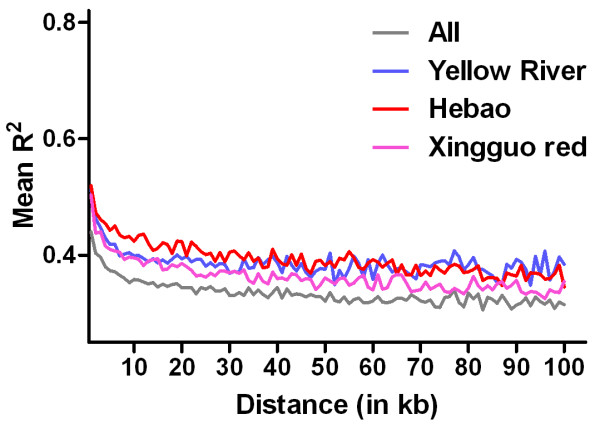
**Decay of linkage disequilibrium (LD) among all samples and three domesticated strains.** LD decay within a range of 100 kb was plotted on all samples and three domesticated strains. Average r^2^ value of each 1 kb region was calculated (Y axis), and physical distances of SNPs was assigned to X axis in unit of kb. X-Y plots were drawn among all samples (grey), within Hebao carp (red), within Yellow River carp (blue), and within Xingguo red carp (purple).

The extent of LD across the SNPs that are on the array was analyzed for all the samples of *C. carpio* and for three of the domesticated strains, Yellow River carp, Hebao carp, and Xingguo red carp. Pairwise r^2^ was calculated using 82,113 SNP markers with MAFs over 0.05 for 120,395 samples for Yellow River carp, 73,703 for Hebao carp, and 86,517 for Xingguo red carp. The average r^2^ within each kilo base pair was calculated and plotted against the physical distance (Figure 5). A similar trend of LD decay was observed in all samples and in each strain, showing that the LD blocks in *C. carpio* are shorter than most other species [40-45]. On the other hand, the LD blocks in these three strains are relatively longer than the LD blocks in all the samples tested, probably because of simpler genetic background within each strain. Similar results have been reported in other species; for example, the domestic dog in which much longer LD blocks have been reported in each breed compared with in mixed samples [44]. In a future study, we will use larger samples of each strain for LD analysis and construct haplotypes, which will be useful for the design of medium or low density SNP panels. As observed previously in several domesticated animals [46,47], lower density SNP panels can be designed and applied for genomic selection and breeding, with fewer tag markers selected on interesting traits.

### Population structure analysis through identity by state (IBS) clustering

**Figure 6:**
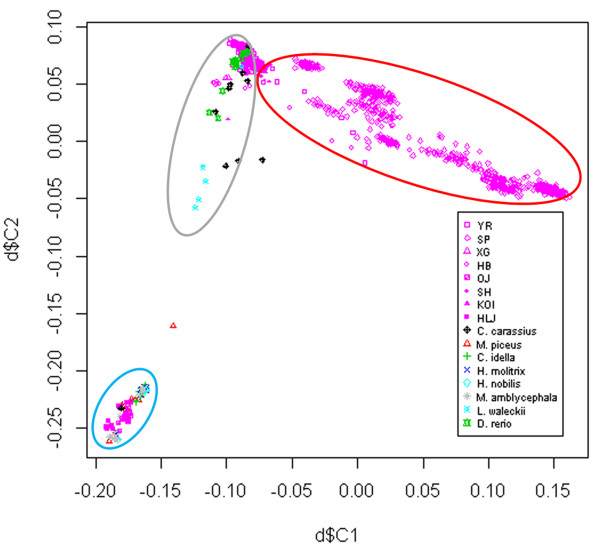
**IBS clustering of all samples.** MDS file was extracted and plotted using R package. The first dimension (d$C1) was assigned to X axis, and the second dimension (d$C2) was assigned to Y axis. Purple symbols represented *C. carpio* samples, and different strains were plotted with different shapes. YR represents Yellow River carp, SP for Songpu mirror carp, XG for Xingguo red carp, HB for Hebao carp, SH for Songhe carp, and KOI for Koi. Symbols with other colors represented other eight species.

Population structure analyses have commonly been conducted before GWAS analyses [48,49] and several methods for population stratification have been developed, such as IBS and principle component analysis (PCA). In this study, genotyping was performed on 1,072 samples of *C. carpio* and on 80 samples of another eight related species. After quality control 73,377 markers and 1,152 samples passed all the criteria. Multidimensional scaling analysis of an IBS matrix revealed the substructure of the samples (Figure 6). All the samples were divided into three clusters. All the *C. carpio* samples (except Oujiang color carp and Heilongjiang carp) formed the largest cluster, within which different strains were grouped together. The Oujiang color carp and Heilongjiang carp genotyping results were both from the first 96-well plate of this array, so a replicate experiment should be performed along with the next batch of samples. *C. carassius, D. rerio* and *L. waleckii* formed the second cluster, close to the largest cluster. The third cluster consisted of *C. idella*, *M. piceus, H. molitrix, H. nobilis* and *M. amblycephala and* showed distinct divergences from the other two clusters. The IBS clustering results are consistent with several phylogenetic analyses of Cyprinidae reported previously [50-52], indicating that the Carp SNP array is reliable and potentially has applications in breeding.

## Conclusions

We developed the Carp SNP array which is the first high-throughput genotyping platform for *C. carpio*. After evaluation with large samples, nearly three fourths of the designed 250,000 SNPs proved to be polymorphic in *C. carpio*. Besides, the Carp SNP array was also evaluated in related species. LD was calculated and longer haplotype blocks were observed in domesticated strains. IBS was conducted and most of the samples were assigned to different clusters. This study indicates that the Carp SNP array will be valuable for farmed carp and for genetic and population biology studies in *C. carpio* and related species.

## Methods

### Ethics statement

This study was approved by the Animal Care and Use Committee (ACUC) of the Centre for Applied Aquatic Genomics at the Chinese Academy of Fishery Sciences. All sampling procedures complied with the guidelines of ACUC on the care and use of animals for scientific purposes.

### Sample collection and genome re-sequencing

Five strains (here a “strain” is defined as a domestic population with unique characteristics; different strains belong to the same species) of *C. carpio* comprising 18 accessions (here “accession” means individual) were collected. The five strains were Songpu carp from Heilongjiang Fishery Research Institute, Yellow River carp from Henan Academy of Fishery Sciences, Heilongjiang River carp from Fuyuan County in Heilongjiang Province, Hebao carp from Wuyuan County in Jiangxi Province, and Oujiang color carp from Longquan County in Zhejiang Province. Fin chips or blood samples were collected and DNA was extracted using a DNeasy Blood & Tissue Kit (Qiagen, Shanghai, China). The samples are listed in Table 1. DNA library preparation and sequencing were carried out at the HudsonAlpha Genomic Services Laboratory (Huntsville, AL, USA) following the manufacturer’s instructions. After KAPA quantitation and dilution, the library was sequenced on Illumina HiSeq 2000 to generate 101 bp paired-end reads.

### SNP identification

The paired-end reads from each accession were aligned to the reference genome using BWA [53] to generate sequence alignment/map SAM files. After mapping, SNPs were identified on the basis of the mpileup files generated by SAMtools [54]. The variant call format (VCF) files were manipulated further using custom-made scripts for primary filtration based on depth and quality.

### SNP selection

SNP selection was carried out in multiple steps using different criteria. All the filtration parameters were set to minimize the risk of false positive sites and to select SNPs that were relatively evenly distributed across the genome. All the original SNPs were classified to six different databases and selected in a certain order. First, non-synonymous SNPs and SNPs in UTR regions were selected; then other transcriptome SNPs were added; and finally, strain-shared and strain-specific SNPs were added to the pool of candidate SNPs. During the SNP selection steps, several custom-made scripts were used to qualify flanking sequences. To ensure an even distribution of SNPs over the genome, a custom-made algorithm (described below) was used. When a new SNP was introduced into the final pool, a threshold of *t* bases was set and SNPs within the *t* bases were excluded. For SNPs that originated from the transcriptome data, *t* was set lower than 2 kb so that all the cSNPs were included in the final pool. For SNPs from the genome re-sequencing data, *t* was set over 10 kb because most of these SNPs were from non-coding regions.

### Evaluation of the SNP array

To evaluate the Carp SNP array, 1,072 samples from *C. carpio* and 80 samples from carp-related species were collected. Genomic DNA was extracted from blood using a DNeasy 96 Blood & Tissue Kit (Qiagen). All the DNA samples were quantified by NanodropND-1000 spectrophotometer (NanoDrop Technologies Inc., Wilmington, DE, USA) and sent to GeneSeek (Lansing, MI) for genotyping. The genotype data were extracted and converted to Ped/Map format. PLINK software [55] was used to classify the SNPs and extract the data for the different species. Mendelian analysis and LD decay were also conducted with PLINK using the “--mendel” and “--r2” parameters. Mendelian analysis was conducted on family data for two parents and 80 offspring, following the procedure reported previously [56]. X-Y plots were drawn using the average r^2^ values (Y axis) and the physical distances (X axis) for each pair of SNPs each kilo base-pair. IBS clustering was conducted with PLINK using the “--mds-plot 2”, “--cluster”, and “--genome” parameters, with a P-value threshold of 1E-3. The PLINK MDS file was extracted and a scatter plot was drawn using d$C1 (X axis) and d$C2 (Y axis) in the R software package (version 3.0.2, Vienna, Austria).

## Competing interests

The authors declare that they have no competing interests.

## Authors’ contributions

JX worked on sample collection, genome sequencing, SNP identification and evaluation, array design and manuscript preparation. ZZ worked on sample collection and SNP array evaluation. XZ, XZheng, YK, JL, YZ, JF, CL, JY, QL established *C. carpio* families of multiple strains, and collected samples for array analysis. YJ participated in manuscript preparation. QL, YZhu and YLiu worked on DNA extraction. PX conceived and supervised *C. carpio* SNP identification and the array project. XS supervised the *C. carpio* genome project. All authors read and approved the final manuscript.
